# Examining the factors contributing to a reduction in hardship financing among inpatient households in India

**DOI:** 10.1038/s41598-024-57984-1

**Published:** 2024-03-26

**Authors:** Arya Rachel Thomas, T. Muhammad, Santosh Kumar Sahu, Umakant Dash

**Affiliations:** 1 Department of Humanities and Social Sciences, Indian Institute of Technology (IIT), Madras, Chennai, Tamil Nadu 600036 India; 2 Department of Family and Generations, International Institute for Population Sciences, Mumbai, Maharashtra 400088 India; 3 Institute of Rural Management Anand (IRMA), Near NDDB, PO Box-60, Anand, Gujarat 388001 India

**Keywords:** Health care, Health care economics, Health policy

## Abstract

In India, the rising double burden of diseases and the low fiscal capacity of the government forces people to resort to hardship financing. This study aimed to examine the factors contributing to the reduction in hardship financing among inpatient households in India. The study relies on two rounds of National Sample Surveys with a sample of 34,478 households from the 71st round (2014) and 56,681 households from the 75th round (2018). We employed multivariable logistic regression and multivariate decomposition analyses to explore the factors associated with hardship financing in Indian households with hospitalized member(s) and assess the contributing factors to the reduction in hardship financing between 2014 and 2018. Notably, though hardship financing for inpatient households has decreased between 2014 and 2018, households with catastrophic health expenditure (CHE) had higher odds of hardship financing than those without CHE. While factors such as CHE, prolonged hospitalization, and private hospitals had impoverishing effects on hardship financing in 2014 and 2018, the decomposition model showed the potential of CHE (32%), length of hospitalization (32%), and private hospitals (24%) to slow down this negative impact over time. The findings showed the potential for further improvements in financial health protection for inpatient care over time, and underscore the need for continuing efforts to strengthen the implementation of public programs and schemes in India such as Ayushman Bharat Pradhan Mantri Jan Arogya Yojana (PMJAY).

## Introduction

Global health spending peaked at 10.8% of gross domestic product (GDP) in 2022. However, this health spending is strongly biased in favor of rich countries. With 15% of the worldwide population, high-income countries account for 80% of total health expenses. In contrast, lower-middle-income countries, with 43% of the world population, collectively account for only 16% of global health spending^1^. The source of financing for health care also varies with the fiscal capacity of the countries. In high-income countries, government spending remains the primary funding source, whereas, in poorer countries, 44% of health expenditure is financed by individuals^2^. Few people fall into extreme or relative poverty in countries with high public spending^3^. The 2030 sustainable development goal 3.8 refers to the attainment of universal health coverage (UHC), which targets quality health services to everyone while ensuring their financial protection. Therefore, increased government spending is essential to provide equitable access to health care, especially in low- and middle-income countries (LMICs)^1^.

In India, many central government- and state-government-sponsored insurance schemes have been introduced to increase access to health care for the poor and vulnerable populations. One important scheme was the Rashtriya Swasthya Bhima Yojana (RSBY) in 2008^4^. It was aimed at insuring secondary and tertiary health services to the below-poverty-line families and was later extended to the poor families in the informal sector above the poverty line. To mitigate the drawbacks of RSBY, such as low coverage cap, low enrolment, and high out-of-pocket expenditure (OOPE)^5–7^, in 2018, Ayushman Bharat Pradhan Mantri Jan Arogya Yojana (PMJAY) was launched. It subsumed RSBY and the Senior Citizen Health Protection Scheme^8^. PMJAY seeks to address the needs of 40% of the poorest population in India, approximately 107.4 million low-income families, making it the largest such scheme in the world. The scheme provides five lakhs Indian rupees per family annually for secondary and tertiary health care needs through enrolled hospitals^9^. PMJAY has been faring well since its inception in 2018. However, evidence shows that government expenditure on health should be at least 5% of the country’s GDP to move towards UHC^10^. In India, in the financial year 2019, government health expenditure reached only 1.3% of GDP^11^. Moreover, the ability of publicly funded health insurance in India to provide financial safety was limited^12,13^.

The shortfalls in government-sponsored health protection can force people to spend out-of-pocket in the event of a health shock. Despite government-sponsored insurance in India, health expenditure impoverishes poor and near-poor households^7^. Studies point out that in LMICs, the direct and indirect cost of health care has severe economic consequences on households, like catastrophic health expenditure (CHE), which forces the households to reduce their consumption level and increase the likelihood of borrowing and selling^14,15^**.**

Moreover, India experiences a double burden of diseases^16^, i.e., the epidemiological shift led to a steep rise in non-communicable diseases (NCD) in the country, along with a pre-existing set of communicable diseases. Further, people lack trust in the public health system^12^ and the utilization of expensive private health facilities is higher in the country, irrespective of the economic status of the households^12,17,18^. Along with the ineffectiveness of public-funded health insurance in combating health expenditure, the large burden of diseases and lack of trust in public hospitals worsened the household's economic status and forced them to borrow or sell to meet health expenditures^13,18^.

### Coping with the health care costs for chronic diseases

Households use various financial coping strategies like borrowing, depletion of assets, dissaving, etc., to safeguard their current consumption levels from economic shocks related to illnesses^19–21^. Nevertheless, some past works revealed the limitations of these informal coping strategies in smoothening consumption, especially for severe illnesses^22,23^. Studies in Indonesia show evidence of imperfect consumption smoothing during illness shocks^24^, mainly in rural and poorest quartile households^25^. Similar results of lowered consumption due to illness shocks are observed among rural households in a low-income setting of Ethiopia^26^. In India, 44% of households in urban areas and 52% in rural areas rely on savings, borrowings, selling assets, and transferring finances for inpatient care needs^27^. However, these people resort to mechanisms other than dissaving when they are too poor to afford health expenditure out of current income or savings or cannot give up any more current consumption owing to poverty. Although savings could be a solution to the extensive medical expenses, in developing countries, they are low and often seen as less stressful to the populace than borrowing at a high interest rate or selling assets^28,29^. Indigent households in some areas of India, whose daily income is less than 1–2$, spend approximately 4% monthly on informal credit^30^. As such, hardship financing refers to these informal coping strategies that households use to protect themselves against financial shocks from illnesses.

### Improvements in the health system for inpatient care

In Asia, between 2015 and 2017, there was a reduction in the proportion of people who fell into relative poverty due to OOPE0^3^. In India, the OOPE as a percentage of total health expenditure has decreased to 48.2% in the financial year 2019 from 64.2% in the financial year 2014^11^. The literature points out an increase in the utilization of public healthcare facilities from 2014 to 2017–18 in India and a consequent decline in OOPE and CHE^31^. Nonetheless, people are pushed into financial catastrophe owing to inpatient expenditures^32^. The poor and near-poor people face financial hardship even when the OOPE is less than 10% of household income^3^. When encountering income losses, poor households often turn to selling off their productive assets or incurring debt. As documented, using hardship financing to investigate impoverishment can help overlook some CHE limitations^28^.

Most studies that looked into hardship financing in India in the past have either been disease-specific, state-specific or cross-sectional studies^18,29,33–37^. In India, the majority of government schemes focus on providing financial protection for inpatient care, and government expenditure on health for inpatient care is always higher than outpatient care^38^. Evidence from a recent study indicates a decrease in hardship financing for inpatient care from 2014 to 2017–2018^13^. However, a decline in hardship financing does not mean that hardship financing has ceased. The reduction in hardship financing is an ongoing process, and at every stage, it is important to assess how each underlying factor has contributed to the decline. Therefore, an in-depth investigation to know the contributing factors to this decline is critical. It is required to understand the implications of existing government interventions and discover a further action plan to eliminate hardship financing. The present study is a novel attempt to identify the contribution of various socioeconomic and demographic factors to the change in hardship financing from 2014 to 2018 for inpatient households. This would help us determine the impact of various underlying factors on the change in hardship financing over time and what must be done to combat health-related expenditures further. Thus, the objectives of the present study are (1) to explore the factors associated with hardship financing in Indian households with hospitalized member(s) and (2) to assess the contributing factors to the reduction in hardship financing from 2014 to 2018.

The current study is divided into four sections. The methods section discusses the details of the data used in the study and the statistical methods used to decompose the impact of underlying factors on the reduction in hardship financing in India. The results section presents the interpretation of the results, followed by a discussion section that elaborates on our significant findings on what and how various factors contribute to the reduction in hardship financing and compares between existing studies and suggests policy implications of the current findings. The final part of the paper reports concluding remarks from the current findings on factors influencing the reduction in hardship financing among inpatient households in India.

## Methods

### Data

The study relied on two rounds of repeated cross-section National Sample Surveys (NSS), namely Survey on Social Consumption (71st round; 2014) and Social Consumption in India: Health (75th round; 2017–18) by the National Sample Survey Organization (NSSO), Ministry of Statistics & Programme Implementation, Government of India. NSS is a large-scale, nationally representative data that provides unit-level information. A stratified multi-stage sampling method for data collection was used to ensure that the sample data will represent the whole nation. The data includes household and individual-level information on their socioeconomic status, morbidity indicators, government-sponsored health insurance schemes, utilization of health services, and health expenditures associated with inpatient and outpatient services. Additional information regarding the survey design and data collection is available in the survey reports^39,40^.

For the present study, we used the inpatient NSSO data, which provides detailed information on hospitalization cases 365 days prior to the interview. The sample survey entailed individual-level information on 57,546 hospitalization cases in 2014 and 93,925 hospitalization cases in 2018, and the entire survey covered 65,932 households in 2014 and 113,823 households in 2018. The current analysis was conducted at the household level, and we limit the study to households with at least one ill individual who has sought inpatient hospitalization services. We focus on studying the impact of unforeseen health shocks on hardship financing and, therefore, exclude hospitalization cases for childbirth from the study. Thus, in our analyses, we used a sample of 34,478 households with at least one inpatient from the 71st round (2014) and 56,681 households with at least one inpatient from the 75th round (2018).

## Measures

### Outcome variable

The outcome variable of our study was hardship financing of the inpatient households. NSSO 71st and 75th round questionnaires asked the respondents to report the source of funds for household health expenditure (excluding any reimbursement) during hospitalization. The possible responses were coded as 1 if the money was funded through household income or savings, 2 if they had borrowed money, 3 if they sourced the fund by selling physical assets such as cattle or jewellery, 4 if the money came as contributions from friends or relatives, 5 if there are any other sources of financing^39,40^. An inpatient household in this study was identified as having hardship financing if it resorts to any financial coping strategies of borrowing, selling of physical assets, contribution from friends or relatives or any other source to meet the health care expenditure of at least one of its members^18,28^. We have coded it as 0 (if the inpatient household does not resort to hardship financing) and 1 (if the inpatient household resorts to hardship financing).

### Explanatory variables

Various socioeconomic, demographic and health characteristics that have been shown to impact household hardship financing have been used in this study^13,29,41^.

### Household-related variables

The age group to which the household head belongs to was recoded as 0 = working-age adult (18–59 years) and 1 = older adult (60 + years), sex of the household head was recoded as 0 = male head 1 = female head, the household composition was recoded into categories 0 = household with children and adult only, 1 = household with elderly and adults only, 2 = household with only adults, 3 = household with children, adults and elderly, 4 = household without adults, household sex composition was recoded as 0 = households with both women and men or only men and 1 = household with only women.

The employment status of the household head was categorized as 0 = self-employed, 1 = regular wage, 2 = casual labour, 3 = no income, living condition index was coded as 0 = low living condition index, and 1 = high living condition index, household expenditure was used to categorize households based on their economic status. It was recoded as 0 = quintile one, 1 = quintile two, 2 = quintile three, 3 = quintile four and 4 = quintile five, CHE of the household was coded as 0 = household without CHE, 1 = household with CHE.

Area of residence was recoded as 0 = rural and 1 = urban, geographical location was recoded as 0 = north zone, 1 = east zone, 2 = west zone, 3 = central zone, 4 = north-east zone and 5 = Union territory, Social groups were categorized as other castes = 0, scheduled caste (SC)/scheduled tribe (ST) = 1, and other backward castes ( OBC) = 2, Education status of the household head was recoded as 0 = illiterate, 1 = education less than primary school, 2 = completed primary, 3 = middle school, 4 = completed secondary or higher secondary and 5 = graduation and above.

### Health-related variables

Insurance status was recoded as a household with no member having insurance = 0 and at least one member having insurance = 1, death of a household member was recoded as 0 = all members are alive and 1 = household with at least one deceased member, chronic disease status was recoded as 0 = no member of household suffered from chronic disease and 1 = at least one member suffered from chronic illness, type of medical facility used was recoded as 0 = only public facilities and 1 = at least one patient member used private facilities, NCD status of household was recoded as 0 = household with one inpatient member suffering from NCD only, 1 = household with more than one inpatient member suffering from NCD, 2 = household with one inpatient suffering from non-NCD alone, 3 = household with more than one inpatient suffering from non-NCD and 4 = households with inpatient(s) suffering from both NCD and non-NCD, number of days hospitalized was categorized as 0 = up to 4 days, 1 = 5–10 days, 2 = more than 10 days up to a year. An exploratory analysis based on the number of patients in each group was used to categorize the length of hospitalization^42,43^.

The diseases were categorized into NCD, communicable and other diseases based on categorization followed by previous literature^44,45^. We have re-categorized them into NCD and non-NCDs, where non-NCDs comprise communicable and other diseases. Chronic ailments reported in NSSO 75th round were severe ailments affecting any of the organs in the person’s body and have significant symptoms lasting for more than a month. Factor analysis using principal component analysis for factor extraction was conducted based on household-level information on the kind of energy used in households, the types of drainage systems, latrine systems, and drinking water, and arrangements for garbage disposal to measure the living condition index^13,46,47^. This was following the Indian Demographic and Health Surveys (DHS) that have employed principal component analysis to construct wealth indices with a set of binary variables on various asset ownerships^48^. Before conducting the factor analysis, we conducted the Kaiser–Meyer–Olkin Measure of Sampling Adequacy (within acceptable range) and Bartlett's test for sphericity (significant at 1% level of significance). The eigenvalues and proportion of variations explained by each factor are given in the appendix (see Appendix Table 1). Households’ OOPE on health is catastrophic when the health expenditure exceeds an arbitrarily set threshold of the total household expenditure^27,49–51^. The different thresholds and the percentage of people suffering CHE at all these thresholds are given in Table 2 of the Appendix. The official CHE threshold for measuring universal health coverage financial protection are 10% and 25%, of which, the 10% threshold is the most commonly used^52^. In India, around 30% of inpatient households sacrificed more than 10% of total household expenditure to meet their health expenditures^27^. Based on evidence from these previous studies, we kept the threshold for CHE as 10% of the total household expenditure^27,53^. Since we are using the information on household expenditure in two periods, to adjust for inflation, we have adjusted the 2018 prices using the annual average consumer price index^54^.

**Table 1:** Descriptive statistics of household characteristics in 2014 and 2018.

Variables	2014		2018	
	N	Weighted percentage	N	Weighted percentage
Age group of household head				
Working age adult	25,745	74.26	43,286	76.41
Older adults	8733	25.74	13,395	23.59
Sex of the head				
Male	30,451	88.43	50,371	89.06
Female	4027	11.57	6310	10.94
Household composition				
Children and adults only household	15,272	42.98	23,876	41.94
Older adults and adult household	3905	11.48	6614	11.85
Only adult household	6769	19.09	14,043	23.71
Children, adults and elderly household	7412	22.40	10,307	18.96
Households without adults	1120	4.05	1841	3.54
Sex composition of household				
With only women	784	2.55	1226	2.21
Other households	33,694	97.45	55,455	97.79
Education of head				
With no formal education	9233	30.15	13,529	27.10
Education less than primary	3869	11.89	5364	10.38
Completed primary	4754	13.72	7298	13.81
Middle school	5542	15.79	8905	15.65
Completed secondary or higher secondary	7504	20.15	14,617	23.34
Graduation and above	3576	8.30	6968	9.71
Quintile				
First quintile	5640	17.30	9622	19.23
Second quintile	7793	22.78	9046	17.48
Third quintile	4497	13.35	12,516	22.59
Fourth quintile	7510	21.84	10,161	17.30
Fifth quintile	9034	24.72	15,336	23.40
Insurance coverage				
No household member is insured	27,102	76.60	43,444	76.63
One or more members insured	7376	23.40	13,237	23.37
Death of any household member				
All members are alive	32,388	93.53	54,536	95.84
At least one dead member in the last year	2090	6.47	2145	4.16
Area of residence				
Rural	18,773	65.55	31,677	65.02
Urban	15,705	34.45	25,004	34.98
Geographical region				
North zone	6384	17.56	10,577	19.59
South zone	7484	32.08	12,623	28.81
East zone	6235	19.45	9587	20.31
West zone	6127	20.13	9516	19.76
Central zone	2466	6.75	3981	6.28
North-east zone	3972	2.16	7021	2.43
Union Territory	1810	1.87	3376	2.83
Social group				
SC/ST	9706	25.63	16,217	25.4
OBC	13,612	44.42	22,562	43.43
Others	11,160	29.95	17,902	31.17
Medical facility used				
Public	15,037	38.35	26,328	41.6
Private	19,309	61.65	30,196	58.4
Disease type				
One inpatient has NCD only	14,187	40.78	23,624	40.76
More than one inpatient having NCD only	565	1.79	591	1.12
One inpatient member has non-NCD only	17,653	50.35	30,101	52.81
More than one inpatient having non-NCD	1009	3.49	1235	2.8
Inpatients with both NCD and non-NCD	1061	3.59	1130	2.5
Living condition index				
Low	17,414	57.09	31,977	63.68
High	17,064	42.91	24,703	36.32
Number of days hospitalized				
Up to 4 days	15,434	45.93	29,541	51.33
4 to 10 days	13,073	36.89	19,746	35.71
More than 10 days to a year	5970	17.17	7394	12.96
CHE				
No	18,735	53.41	33,398	56.44
Yes	15,743	46.59	23,283	43.56
Chronic diseases				
Not suffered	24,880	67.79	45,126	75.50
Suffered	9598	32.21	11,555	24.50

*N* Number of observations in the sample.

**Table 2:** Prevalence of hardship financing by background characteristics of hospitalized households.

Variables	2014	2018	Difference	*p* value
	Weighted percentage	Weighted percentage		
Age group of household head				
Working-age adult	29.23	18.17	− 11.06	< 0.001
Older adults	28.86	19.76	− 9.10	< 0.001
Sex of the head				
Male	28.75	17.65	− 11.10	< 0.001
Female	32.10	25.82	− 6.28	< 0.001
Household composition				
Children and adult only household	29.39	17.75	− 11.64	< 0.001
Older adult and adult only household	29.51	18.85	− 10.66	< 0.001
Only adult household	30.67	19.38	− 11.29	< 0.001
Children, adult and elderly household	26.25	16.00	− 10.25	< 0.001
Household without adult	34.16	34.97	0.81	< 0.050
Sex composition of household				
With only women	28.9	18.19	− 10.71	< 0.001
Other households	38.01	34.52	− 3.49	< 0.001
Education of head				
No formal education	36.95	23.79	− 13.16	< 0.001
Education less than primary	34.91	20.31	− 14.6	< 0.001
Completed primary	30.76	20.67	− 10.09	< 0.001
Middle school	27.39	16.57	− 10.82	< 0.001
Completed secondary or higher secondary	21.17	15.32	− 5.85	< 0.001
Graduation and above	12.45	9.95	− 2.5	< 0.100
Quintile				
First quintile	33.92	23.2	− 10.72	< 0.001
Second quintile	32.62	21.04	− 11.58	< 0.001
Third quintile	32.45	18.86	− 13.59	< 0.001
Fourth quintile	28.51	18.77	− 9.74	< 0.001
Fifth quintile	21.34	12.40	− 8.94	< 0.001
Insurance coverage				
No household member is insured	26.99	16.48	− 10.51	< 0.001
One or more members insured	36.16	25.34	− 10.82	< 0.001
Death of any household member				
All members are alive	28.76	18.16	− 10.6	< 0.001
At least one dead member in the last 1 year	34.63	27.5	− 7.13	< 0.001
Area of residence				
Rural	31.69	19.95	− 11.74	< 0.001
Urban	24.27	15.94	− 8.33	< 0.001
Geographical region				
North zone	22.98	17.71	− 5.27	< 0.001
South zone	42.8	26.42	− 16.38	< 0.001
East zone	29.69	17.23	− 12.46	< 0.001
West zone	18.49	12.87	− 5.62	< 0.001
Central zone	22.99	17.37	− 5.62	< 0.001
North-east zone	7.03	7.13	0.10	< 0.001
Union Territory	9.18	5.72	− 3.46	< 0.001
Social group				
SC/ST	32.60	19.59	− 13.01	< 0.001
OBC	31.84	19.89	− 11.95	< 0.001
Others	22.17	15.83	− 6.34	< 0.001
Medical facility used				
Public	22.22	14.83	− 7.39	< 0.001
Private	33.38	21.18	− 12.2	< 0.001
Disease type				
One inpatient has NCD only	32.32	22.22	− 10.10	< 0.001
More than one inpatient having NCD only	45.19	30.62	− 14.57	< 0.050
One inpatient member has non-NCD only	24.39	14.81	− 9.58	< 0.001
More than one inpatient having non-NCD	35.61	22.93	− 12.68	< 0.001
Inpatients with both NCD and non-NCD	45.19	27.21	− 17.98	< 0.001
Living condition index				
Low	33.62	20.6	− 13.02	< 0.001
High	23.18	14.96	− 8.22	< 0.001
Number of days hospitalized				
Up to 4 days	18.6	13.66	− 4.94	< 0.001
4 to 10 days	65.59	20.41	− 45.18	< 0.001
More than 10 days to a year	54.01	32.77	− 21.24	< 0.001
Catastrophic health expenditure				
No	16.47	11.46	− 5.01	< 0.001
Yes	43.65	27.75	− 15.90	< 0.001
Chronic diseases				
Not suffered	26.59	16.6	− 9.99	< 0.001
Suffered	34.50	24.55	− 9.95	< 0.001

The *p* value reported is based on the proportion test.

### Statistical analysis

The statistical analysis of the data was conducted on Stata version 15.1, and the multivariate decomposition analysis was conducted using the command *mvdcmp*. Sampling weight was used for reporting weighted percentages in descriptive statistics, logistic regression, and decomposition analysis. De-normalized weights were used in the logit decomposition analysis using the pooled data from the period 2014 and 2018^47,55^.

Descriptive statistics were reported to understand the general distribution of samples. The prevalence of hardship financing was calculated for both periods. A proportion test was conducted, and p values were reported to show the level of significance of the change in hardship financing between 2014 and 2018. Multivariable logistic regression analyses were carried out to find the significant factors associated with hardship financing in households in 2014 and 2018.

The logistic regression can be summarized in the following Eq. (1):

1 $$Ln\left( {\frac{{p_{i} }}{{1 - p_{i} }}} \right) = \beta_{0} + \beta_{1} X_{ij} + \beta_{2} Y_{ij} + \beta_{3} Z_{ij} + \epsilon_{0}$$

where $p_{i} =$ Probability of success or Hardship financing (coded as1), $1 - p_{i} =$ Probability of failure or no Hardship financing (coded as 0), $X_{ij} =$ Household disease composition, categorized as: 1 = Household with one member having only NCD, 2 = Household with more than one member having only NCD, 3 = Households with one member have only non-NCD, 4 = Household with more than one member having only non-NCD, 5 = Households with both NCD and non NCD, $Y_{ij} =$ Vector of individual characteristics, $Z_{ij} =$ Vector of household characteristics, $\epsilon_{0} =$ Error term.

Furthermore, a decomposition analysis identified the crucial factors that led to the change in hardship financing between 2014 and 2018. The Blinder and Oaxaca models of decomposition developed in the early 1970s have been used for decomposing linear regression models^56,57^. Multivariate decomposition analysis is extended to non-linear regression decomposition models and can be used for linear, count or logit models^58^. Throughout literature studies have used multivariate decomposition analysis to study the contribution of underlying factors in non-linear models with a time component^59–62^. Such a decomposition analysis will help us to quantify the contributions of underlying factors to change in hardship financing over time. Our model is summarised as follows:

2 $$Logit\left( {Y_{t1} } \right) - Logit\left( {Y_{t2} } \right) = \underbrace {{\left\{ {\overline{{F\left( {X_{t1} \beta_{t1} } \right)}} - \overline{{F\left( {X_{t2} \beta_{t1} } \right)}} } \right\}}}_{{\text{E}}} + \underbrace {{\left\{ {\overline{{F\left( {X_{t2} \beta_{t1} } \right)}} - \overline{{F\left( {X_{t2} \beta_{t2} } \right)}} } \right\}}}_{{\text{C}}} .$$

In Eq. (2) mentioned above, time t1 (2014) is the comparison group, and t2 (2018) is the reference group. X represents the vector of dependent variables and $\beta$ vector of regression coefficients. The mean difference in Y between 2014 and 2018 can be decomposed into a characteristics effect and a coefficient effect. The E component captures the differential in two time periods due to differences in the endowments or characteristics. This is the explained component of the model, also called the characteristics effect. The C refers to the differential in the two time periods due to differences in coefficients or the effects. This is the unexplained component of the model, also called the coefficient effect. A positive E coefficient would indicate the reduction in hardship financing for households with hospitalization cases between the two time periods if the distribution of X between the two time periods were equal. At the same time, a negative C coefficient indicates an increase in hardship financing between the two time periods if both periods have the same behavioral responses^58^.

The mean–variance inflation factor of the logistic regression models was less than two and shows no evidence of multicollinearity. We checked for the robustness of the logistic models of $t_{1}$ (2014) and $t_{2}$ (2018) using the pooled cross-section regression model combining the information from both periods. The outcome of pooled regression was comparable to the results of $t_{1}$ and $t_{2}$ models, thereby indicating the relevance of each independent variable used in the analyses.

## Results

Table 1 shows the profile of the sample population in 2014 and 2018. Out of the total households, 25.74% and 23.59% were headed by older adults in 2014 and 2018, respectively. Most of the households in both survey rounds were male headed. In 2018, 3.54% of the sample households had only older adults, whereas 23.71% had only adults. Similarly, 2.21% of the sample households had only female members. Further, around 27.1% of the households were headed by members without formal education, whereas 9.71% of household heads had a higher education (graduation and above). Around 23.37% of the households had at least one member covered by health insurance, and 24.5% had a chronic disease patient in 2018, which was even higher (32.21%) in 2014.

**Figure 1:**
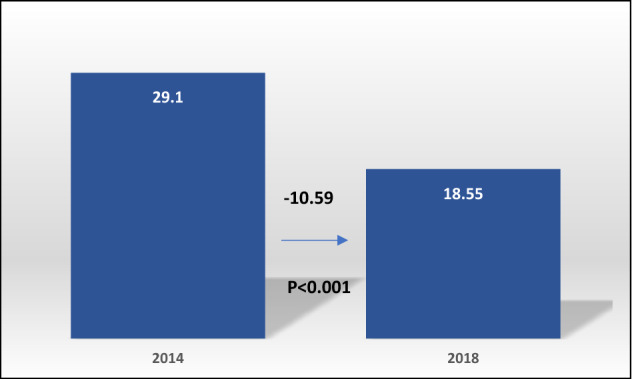
Change in the prevalence of hardship financing (weighted percentages) between 2014 and 2018 (significant at *p* < 0.001).

Figure 1 presents the change in the prevalence of hardship financing from 2014 to 2018. Hardship financing was significantly reduced from 29.10% in 2014 to 18.55% in 2018 (Difference: − 10.59%, *p* < 0.001). Table 2 presents the prevalence of hardship financing by the background characteristics in 2014 and 2018, the differences, and their statistical significance.

Table 3 provides the adjusted estimates from the logistic regression of factors associated with hardship financing, separately for 2014, 2018 and the pooled data. In the pooled data, households with a head in the age group of 60 and above were less likely to have hardship financing [AOR 0.88, CI 0.76–1.02] than those with a head in the age group of less than 60. In comparison to those households with children and adults only, households with only adults [AOR 0.90, CI 0.81–1.00] or adults and older adults [AOR 0.82, CI 0.69–0.96] or those with children, adults and older adults [AOR 0.83, CI 0.72–0.96] had lower odds of hardship financing in the pooled data. Further, households with only female members had higher odds of hardship financing [AOR 1.35, CI 1.01–1.80] than other households. Higher levels of education reduced the odds of hardship financing. Households with a head with higher education had lower odds of hardship financing [AOR 0.33, CI 0.28–0.39] than those with no formal education. On the other hand, households with health insurance had higher odds of hardship financing [AOR 1.35, CI 1.23–1.49] than those without insurance.

**Table 3:** Estimates from logistic regression of inpatient household characteristics on hardship financing.

Variables	2014		2018		POOLED	
	Odds Ratio	95% CI	Odds Ratio	95% CI	Odds Ratio	95% CI
Age group of household head						
Working-age adult	Ref		Ref		Ref	
Older adults	0.89	(0.74–1.08)	0.85	(0.70–1.03)	0.88*	(0.76–1.02)
Sex of the head						
Male	Ref		Ref		Ref	
Female	0.84*	(0.70–1.01)	1.18*	(0.99–1.40)	0.94	(0.82–1.08)
Household composition						
Children and adult only	Ref		Ref		Ref	
Older adult and adult only household	0.80**	(0.65–0.99)	0.84	(0.66–1.06)	0.82**	(0.69–0.96)
Only adult household	0.90	(0.78–1.03)	0.90	(0.79–1.02)	0.90**	(0.81–1.00)
Children, adult and elderly household	0.84*	(0.70–1.01)	0.82**	(0.68–0.98)	0.83**	(0.72–0.96)
Household without adult	0.74*	(0.53–1.02)	1.66***	(1.23–2.24)	0.95	(0.74–1.22)
Sex composition of household						
Other households	Ref		Ref		Ref	
With only women	1.39*	(0.97–1.99)	1.40**	(1.00–1.94)	1.35**	(1.01–1.80)
Education of head						
With no formal education	Ref		Ref		Ref	
Education less than primary	0.88	(0.74–1.05)	0.89	(0.75–1.06)	0.89*	(0.78–1.02)
Completed primary	0.81***	(0.69–0.94)	0.89	(0.77–1.03)	0.83***	(0.74–0.93)
Middle school	0.69***	(0.59–0.81)	0.70***	(0.60–0.82)	0.69***	(0.61–0.78)
Completed secondary or higher secondary	0.50***	(0.42–0.60)	0.64***	(0.55–0.74)	0.54***	(0.48–0.61)
Graduation and above	0.29***	(0.23–0.37)	0.42***	(0.33–0.52)	0.33***	(0.28–0.39)
Quintile						
Fifth quintile	Ref		Ref		Ref	
First quintile	1.45***	(1.19–1.75)	1.45***	(1.19–1.77)	1.42***	(1.22–1.64)
Second quintile	1.46***	(1.24–1.72)	1.44***	(1.21–1.73)	1.43***	(1.26–1.63)
Third quintile	1.29***	(1.08–1.55)	1.33***	(1.14–1.55)	1.30***	(1.14–1.48)
Fourth quintile	1.35***	(1.14–1.59)	1.37***	(1.18–1.59)	1.34***	(1.18–1.52)
Insurance coverage						
No member is insured	Ref		Ref		Ref	
One or more members insured	1.26***	(1.12–1.43)	1.59***	(1.41–1.79)	1.35***	(1.23–1.49)
Death of any household member						
All members are alive	Ref		Ref		Ref	
At least one dead member in the last 1 year	1.15	(0.91–1.44)	1.38***	(1.09–1.74)	1.20*	(1.00–1.44)
Area of residence						
Rural	Ref		Ref		Ref	
Urban	1.05	(0.93–1.19)	1.08	(0.96–1.22)	1.06	(0.96–1.17)
Geographical region						
West zone	Ref		Ref		Ref	
North zone	1.14	(0.95–1.37)	1.30***	(1.10–1.54)	1.19**	(1.03–1.36)
South zone	3.28***	(2.83–3.80)	1.95***	(1.68–2.27)	2.78***	(2.49–3.11)
East zone	1.93***	(1.64–2.27)	1.32***	(1.11–1.57)	1.71***	(1.51–1.94)
Central zone	1.14	(0.87–1.49)	1.28*	(0.97–1.70)	1.16	(0.93–1.43)
Northeast zone	0.45***	(0.34–0.60)	0.66***	(0.51–0.86)	0.51***	(0.42–0.63)
Union Territory	0.65**	(0.47–0.92)	0.67	(0.31–1.42)	0.67**	(0.47–0.96)
Social group						
Others	Ref		Ref		Ref	
SC/ST	1.38***	(1.19–1.60)	1.14*	(0.99–1.31)	1.30***	(1.16–1.46)
OBC	1.18**	(1.04–1.34)	1.06	(0.94–1.21)	1.14***	(1.03–1.26)
Medical facility used						
Public	Ref		Ref		Ref	
Private	1.50***	(1.33–1.70)	1.23***	(1.09–1.39)	1.41***	(1.28–1.55)
Disease type						
One inpatient has non-NCD only	Ref		Ref		Ref	
One inpatient has NCD only	1.15**	(1.03–1.29)	1.20***	(1.08–1.33)	1.15***	(1.06–1.26)
More than one inpatient having NCD only	1.53***	(1.12–2.09)	1.37**	(1.01–1.86)	1.49***	(1.16–1.90)
More than one inpatient having non-NCD	1.24	(0.95–1.62)	1.23	(0.90–1.68)	1.23*	(0.99–1.52)
Inpatients with both NCD and non-NCD	1.40**	(1.08–1.80)	1.28*	(0.99–1.65)	1.38***	(1.13–1.68)
Living condition index						
Low living condition index	Ref		Ref		Ref	
High living condition index	0.65***	(0.57–0.75)	0.78***	(0.69–0.88)	0.68***	(0.62–0.76)
Number of days hospitalized						
Less than 5 days	Ref		Ref		Ref	
5–10 days	1.61***	(1.43–1.82)	1.12*	(1.00–1.25)	1.44***	(1.31–1.57)
More than 10 days	2.20***	(1.87–2.58)	1.83***	(1.59–2.11)	2.07***	(1.83–2.34)
CHE						
No	Ref		Ref		Ref	
Yes	3.17***	(2.80–3.60)	2.25***	(2.00–2.53)	2.85***	(2.59–3.14)
Chronic disease						
Not suffered	Ref		Ref		Ref	
Suffered	1.23***	(1.10–1.39)	1.33***	(1.18–1.50)	1.26***	(1.15–1.38)
Year						
2014					Ref	
2018					0.57***	(0.53–0.62)
Constant	0.06***	(0.05–0.08)	0.06***	(0.05–0.08)	0.08***	(0.06–0.10)
Observations	34,338		56,523		90,861	

Odds ratios are adjusted for all the selected covariates.

Ref., Reference category; 95% CI, Robust confidence interval in parentheses.

***p < 0.01; **p < 0.05; *p < 0.1.

Notably, households with a member utilizing private health facilities had higher odds of hardship financing [AOR 1.41, CI 1.28–1.55] than those with members using public health facilities. Households with more than one inpatient member suffering from NCDs had higher odds of hardship financing [AOR 1.49, CI 1.16–1.90] than non-NCDs. On the other hand, households with a high living condition index value were less likely to have hardship financing [AOR 0.68, CI 0.62–0.76] than those with a low living condition. Also, households of members with hospital admissions of more than 10 days had higher odds of hardship financing [AOR 2.07, CI 1.83–2.34] compared to those with less than 5 days. Households with a CHE had higher odds of hardship financing [AOR 2.85, CI 2.59–3.14] than those without CHE. Finally, households with at least one member suffering from a chronic disease were more likely to have hardship financing [AOR 1.26, CI 1.15–1.38] than those without a chronic disease patient.

The estimates from the decomposition analysis (Table 4) suggest that the differences in effects (due to coefficients, C) account for 77.07% of the observed differences (decrease) in the prevalence of hardship financing. However, only 17% of the reduction in hardship financing was explained by differences in compositional characteristics (due to characteristics, E). The E reflects the counterfactual comparison of the difference in outcomes from 2014, i.e., the expected difference if households in 2014 were given a distribution of covariates similar to those of households in 2018. Similarly, C reflects a counterfactual comparison of outcomes from the perspective of households in 2018, i.e., the expected difference if households in 2018 experienced behavioural responses to covariates of those of households in 2014.

**Table 4:** Estimates from multivariate decomposition analysis of reduction in hardship financing.

Variables	Difference due to characteristics (E)			Difference due to coefficients (C)		
	Coeff [95% CI]	Relative contribution	Total contribution (percentage)	Coeff [95% CI]	Relative contribution	Total contribution (percentage)
Age group of household head						
Working-age adult	Ref		− 0.48	Ref		2.31
Older adult	0.0005 [− 0.0001,0.0011]	− 0.48		− 0.0025 [− 0.0170,0.0121]	2.31	
Sex of the head						
Male	Ref		0.14	Ref		− 7.57
Female	− 0.0002 [− 0.0003,0.0000]	0.14		0.0080** [0.0019,0.0141]	− 7.57	
Household composition						
Children and adult only	Ref		0.14	Ref		− 6.47
Older adult and adult only household	− 0.0001 [− 0.0002,0.0000]	0.08		0.0010 [− 0.0065,0.0086]	− 0.98	
Only adult household	− 0.0007 [− 0.0015,0.0002]	0.65		0.0002 [− 0.0073,0.0076]	− 0.16	
Children, adult and elderly household	0.0010* [0.0001,0.0019]	− 0.94		− 0.0012 [− 0.0132,0.0108]	1.11	
Household without adult	− 0.0004** [− 0.0006, − 0.0001]	0.35		0.0068*** [0.0031,0.0106]	− 6.44	
Sex composition of household						
Other households	Ref		0.16	Ref		− 0.02
With only women	− 0.0002* [− 0.0003, − 0.0000]	0.16		0.0000 [− 0.0026,0.0026]	− 0.02	
Education of head						
With no formal education	Ref		3.33	Ref		− 18.43
Education less than primary	0.0003 [− 0.0001,0.0007]	− 0.25		0.0004 [− 0.0058,0.0065]	− 0.33	
Completed primary	0.0000 [− 0.0000,0.0000]	0.01		0.0027 [− 0.0033,0.0087]	− 2.55	
Middle school	0.0001*** [0.0000,0.0001]	− 0.05		0.0002 [− 0.0071,0.0075]	− 0.17	
Completed secondary or higher secondary	− 0.0020*** [− 0.0027, − 0.0013]	1.92		0.0102* [0.0006,0.0198]	− 9.65	
Graduation and above	− 0.0018*** [− 0.0023, − 0.0013]	1.70		0.0061* [0.0004,0.0117]	− 5.73	
Quintile						
Fifth quintile	Ref		0.07	Ref		− 1.10
First quintile	0.0010*** [0.0005,0.0015]	− 0.96		0.0002 [− 0.0096,0.0100]	− 0.15	
Second quintile	− 0.0028*** [− 0.0041, − 0.0015]	2.65		− 0.0006 [− 0.0120,0.0108]	0.56	
Third quintile	0.0038*** [0.0018,0.0058]	− 3.59		0.0008 [− 0.0058,0.0073]	− 0.71	
Fourth quintile	− 0.0021*** [− 0.0030, − 0.0011]	1.97		0.0009 [− 0.0092,0.0109]	− 0.80	
Insurance coverage						
No member is insured	Ref		0.03	Ref		− 10.50
One or more members insured	− 0.0000*** [− 0.0000, − 0.0000]	0.03		0.0111** [0.0029,0.0193]	− 10.50	
Death of any household member						
All members are alive	Ref		1.01	Ref		− 2.34
At least one dead member in the last 1 year	− 0.0011** [− 0.0018, − 0.0003]	1.01		0.0025 [− 0.0019,0.0068]	− 2.34	
Area of residence						
Rural	Ref		− 0.06	Ref		− 2.16
Urban	0.0001 [− 0.0000,0.0002]	− 0.06		0.0023 [− 0.0101,0.0146]	− 2.16	
Geographical region						
West zone	Ref		2.79	Ref		39.02
North zone	0.0008** [0.0003,0.0012]	− 0.71		0.0048 [− 0.0042,0.0138]	− 4.55	
South zone	− 0.0031*** [− 0.0038, − 0.0024]	2.94		− 0.0343*** [− 0.0490, − 0.0197]	32.46	
East zone	0.0003** [0.0001,0.0005]	− 0.29		− 0.0152** [− 0.0250, − 0.0054]	14.36	
Central zone	− 0.0002 [− 0.0004,0.0000]	0.17		0.0017 [− 0.0038,0.0071]	− 1.59	
Northeast zone	− 0.0002** [− 0.0003, − 0.0001]	0.15		0.0017 [− 0.0001,0.0034]	− 1.59	
Union Territory	− 0.0006 [− 0.0016,0.0005]	0.53		0.0001 [− 0.0031,0.0033]	− 0.07	
Social group						
Others	Ref		0.14	Ref		18.56
SC/ST	− 0.0001 [− 0.0001,0.0000]	0.05		− 0.0101 [− 0.0210,0.0009]	9.50	
OBC	− 0.0001 [− 0.0003,0.0001]	0.09		− 0.0096 [− 0.0260,0.0068]	9.06	
Medical facility used						
Public	Ref		0.94	Ref		24.26
Private	− 0.0010** [− 0.0016, − 0.0004]	0.94		− 0.0257* [− 0.0481, − 0.0032]	24.26	
Disease type						
One inpatient has non-NCD only	Ref		0.88	Ref		− 2.48
One inpatient has NCD only	− 0.0000*** [− 0.0000, − 0.0000]	0.02		0.0037 [− 0.0092,0.0167]	− 3.54	
More than one inpatient having NCD only	− 0.0003* [− 0.0006, − 0.0000]	0.29		− 0.0004 [− 0.0020,0.0012]	0.38	
More than one inpatient having non-NCD	− 0.0002 [− 0.0005,0.0001]	0.20		− 0.0001 [− 0.0030,0.0029]	0.07	
Inpatients with both NCD and non-NCD	− 0.0004 [− 0.0008,0.0000]	0.37		− 0.0007 [− 0.0033,0.0020]	0.61	
Living condition index						
Low living condition index	Ref		− 2.22	Ref		− 14.75
High living condition index	0.0024*** [0.0012,0.0035]	− 2.22		0.0156 [− 0.0006,0.0318]	− 14.75	
Number of days hospitalized						
Less than 5 days	Ref		3.66	Ref		32.30
5–10 days	− 0.0002 [− 0.0004,0.0000]	0.17		− 0.0277*** [− 0.0407, − 0.0147]	26.19	
More than 10 days	− 0.0037*** [− 0.0046, − 0.0028]	3.49		− 0.0065 [− 0.0142,0.0013]	6.11	
Catastrophic health expenditure						
No	Ref		3.43	Ref		31.39
Yes	− 0.0036*** [− 0.0042, − 0.0031]	3.43		− 0.0332*** [− 0.0508, − 0.0156]	31.39	
Chronic disease						
Not suffered	Ref		3.04	Ref		− 4.95
Suffered	− 0.0032*** [− 0.0045, − 0.0019]	3.04		0.0052 [− 0.0058,0.0163]	− 4.95	
Total		17.00			77.07	
Constant				− 0.0067 [− 0.0801,0.0667]	6.30	

Coeff., Coefficient; Ref., Reference category; 95% CI, Robust confidence interval in parentheses.

***p < 0.01; **p < 0.05; *p < 0.1.

Differences due to characteristics suggest that equalizing the distribution between 2014 and 2018 for education [Secondary or higher (1.92%), and graduation and above (1.7%)], equalizing the living conditions in the households (2.22%), CHE (3.43%) is expected to contribute significantly to the decrease in hardship financing. Differences due to coefficients suggest that equalizing the behavioural responses of two time periods for female headship (− 7.57%), household composition (− 6.47%), education (− 18.43%), presence of insurance coverage (− 10.5%), social groups (− 2.16%), households with non-NCD and NCD patients (− 2.48%), household living conditions (− 14.75%) is expected to contribute to increase in hardship financing. Additionally, differences due to coefficients also suggest that equalizing behavioural responses of two time periods for factors such as CHE (31.39), private sector hospitalization (24.2%) and geographical regions (39.02%) are primary contributors expected to contribute significantly to the decrease in hardship financing in this study.

## Discussion

The study explored the impact of various socioeconomic, demographic and health characteristics on hardship financing among inpatient households in India. We observed an overall reduction in hardship financing from 2014 to 2018 for households with at least one inpatient in India. The prevalence of hardship financing was 29.10% in 2014 and decreased to 18.55% in 2018. This is on par with a previous study that points out a reduction in hardship financing over time for inpatient cases^13^. Further, a previous study showed that large household OOPE forces people to resort to informal coping strategies^63^. Consistent with previous findings, we found that hardship financing is highly prevalent in households with CHE, and it was more than two times higher in both the time periods (2014 and 2018) compared to households without CHE. Besides, the findings from the decomposition analysis indicate that there is a potential to slow down this increase in hardship financing by about 32% by reducing CHE.

Many studies in India suggest an unchanged or even increased financial hardship after being covered by government-sponsored insurance schemes^13,37,64–66^. This could be due to the flaws in the implementation of the schemes and inefficient allocation of healthcare resources to the insurance beneficiaries. We found similar evidence among inpatient households with insurance having a higher prevalence of hardship financing. These households were more likely to resort to hardship financing than those without insurance in both the time periods. This could be explained by the low awareness among the enrolled population. It could also indicate the increased utilization of hospital services by the inpatient household members with insurance coverage due to their greater affordability and accessibility than those that are not covered by any health insurance. These require further research to find out the reasons considering more qualitative and quantitative aspects and the nature of health insurance, public or private and state or central schemes. Findings from decomposition analysis, however, revealed the expected reduction in hardship financing if inpatient households in 2018 had the same insurance coverage status as in 2014. This shows that the hardship financing from being insured reduced between 2014 and 2018 and has implications for policy. The finding on the impact of health insurance coverage on hardship financing is particularly relevant for debate in the context of achieving UHC in the country.

Importantly, hardship financing is higher in households with only women than in households with men and women. This underlines the reality of large mortality rates among women^67–69^. However, this result contradicts a previous study from an individual-level analysis, which points out the lower hardship financing among women than men^13^. This variation in outcome could be attributed to multiple reasons. One can be the discrimination women face in mixed-sex households. Evidence shows that in a household where women and men are suffering from illnesses, the healthcare needs of women are given lesser attention than that of their male counterparts^70,71^. Secondly, Kochar^72^ suggests that the non-disabled male member is essential for households to smooth shocks. The lack of a male member or a male head could have also contributed to the higher hardship financing in households with only women. However, from decomposition analysis, the positive coefficient of female headship indicates the expected reduction in hardship financing between 2014 and 2018 if women in 2018 had the same protective effects of female headship as in 2014. This highlights the need for developing gender-specific economic policies in order to reduce the levels of hardship financing, especially in female headed households.

The age of the household head and their education status had a significant impact on hardship financing. The prevalence of hardship financing increased with the increase in the age of household head and reduced with the increase in education levels. The findings also indicate that households with elderly heads compared to working-age heads are more likely to face hardship financing, and compared to heads with no formal education, those with higher levels of education are less likely to encounter hardship financing. In China, older adults with chronic diseases who are household heads had higher intensity and incidence of CHE^73^. In India, elderly member households had significantly higher health spending than other households^74^. Literature further showed evidence of a decline in health expenditure with higher education of the household head^24,75^. The positive coefficient of education levels from the decomposition analysis also showed the protective effects of education on reducing hardship financing between 2014 and 2018.

Furthermore, there is strong evidence of high OOPE among households with members suffering from NCD in India^76^. However, a dual burden of disease in the case of India worsens the situation and burdens the public health system^77^. In our study, we found evidence of a dual burden of diseases. In 2014, households with more than one NCD and those with both NCD and non-NCD had similar levels of higher prevalence of hardship financing. In both rounds, households with more than one NCD had the highest likelihood of hardship financing, followed by those households where NCD and non-NCD co-exist. Additionally, studies show that an extended period of hospitalization leads to impoverishment in Indian households^78^. Literature suggests that the financial hardship from illnesses worsens with the severity of diseases^13,20,24^. Evidence from the USA shows that diseases lasting more than 100 days negatively impact consumption levels^79^. In the present study, the prevalence and likelihood of hardship financing were higher among households with extended hospitalization periods and chronic diseases.

The prevalence and likelihood of hardship financing were higher in households with patients who visited the private health facilities compared to the public health facilities for hospital care in 2014 and 2018. This could be attributed to the exorbitant charges of private hospitals and the inadequacy of public sector hospitals^75^. The negative coefficient of the utilization of private health facilities from decomposition analysis shows that hardship financing would have increased between 2014 and 2018 if households in 2018 had the same behavioural responses to utilization of private health facilities as in 2014. However, this increase is slowed by an expected potential for hardship financing to reduce by about 24% from 2014 to 2018. Even when complete financial protection against health costs is far-fetched, government-sponsored schemes like RSBY have contributed to some increased access to hospital care, especially through empanelled private hospitals^80^. Since 2018, PMJAY, the world's largest health assurance scheme caters to the needs of the poorest 40% of the population for hospitalization expenditures in secondary and tertiary care through empanelled hospitals^81^. In 2020, 44% of these empanelled hospitals belonged to the private sector^82^. The rising increased access to private hospitals must contribute to the slowing down of increased hardship financing.

This study has several limitations. Firstly, it is limited to households with at least one case of hospitalization. Secondly, we assume that health shocks are severe when they are idiosyncratic and unexpected. Thus, we do not include hospitalization for childbirth in our study. Thirdly, the dependent variable of the study, hardship financing, is binary. Therefore, we fail to capture the extent and depth of each inpatient household’s hardship financing. Fourth, the recall period for inpatient cases is 365 days, and for outpatients, 15 days^39,40^. Therefore, in this study, we could not combine the health expenditure on inpatients and outpatients to calculate the total CHE. Additionally, since the NSSO data we use is pooled cross-sectional, the absence of a time series component does not allow us to test for a causal relationship that could exist between the independent and dependent variables.

### Implications for program and policy

Our findings showed that, even when complete financial protection for health care is yet to be achieved, there are still improvements in health protection for inpatient care over time. The plausible contributors to the decline in hardship financing could be the continuous efforts of the government to improve public-funded health insurance in India. The government’s budgetary allocation and focus on inpatient care is always higher than outpatient care^38^. From RSBY to PMJAY, there has been a significant increase in the target population covered under the health insurance scheme. Moreover, access to private hospitals has increased through empanelled hospitals under these schemes over time. The paper reveals the need to improve the public health system further and make the private sector accessible to people with limited resources. The study also underscores the impact of the rising dual burden of NCDs and non-NCDs on worsening hardship financing. As many NCDs are lifestyle-based diseases, the study urges the government to empower and incentivize people to adopt preventive health measures to improve their quality of life. Approximately 77% of the inpatient households under study are uninsured, and the study’s findings stress the importance of sound health insurance to reduce hardship financing. Government schemes and policies should aim to develop a health system that does not financially drain society's poor and vulnerable section.

## Conclusions

The findings suggest an overall reduction in hardship financing for inpatient households from 2014 to 2018. In a developing country like India, the government plays a significant role in expanding the provision of health care. The findings can help draw a picture of the government’s efforts to combat financial hardship for health. Even though the CHE has impoverishing effects on hardship financing, the study provides evidence of the potential to slow down this negative impact. Similarly, factors such as the length of hospitalization private sector utilization, despite its impoverishing effects on households in both 2014 and 2018, have the potential to decrease its negative impact on hardship financing over time. Our findings urge the policymakers to address the pressing need for designing and regulating a functional health insurance program to maximize the benefit of risk pooling and safeguard the health and welfare of poor households.

### Supplementary Information

Supplementary Information.

## Data Availability

The study uses secondary data available in the public domain. The two data sets used in the study can be downloaded on request using the following links: http://microdata.gov.in/nada43/index.php/catalog/135, http://microdata.gov.in/nada43/index.php/catalog/152
