# Supplementary material for: Examining the factors contributing to a reduction in hardship financing among inpatient households in India
Source: Sci Rep. 2024 Mar 26;14:7164. doi: 10.1038/s41598-024-57984-1 (PMC10965936; doi:10.1038/s41598-024-57984-1)
Supplement: Supplementary file 1 — Supplementary Information. [file 41598_2024_57984_MOESM1_ESM.docx]

Appendix Table 1: Eigenvalues and proportion of variation of each factor from the principal component factor analysis

| Factors | 2014 | | | | 2018 | | | |
| --- | --- | --- | --- | --- | --- | --- | --- | --- |
|  | Eigen value | Difference | Proportion | Cumulative | Eigen value | Difference | Proportion | Cumulative |
| 1 | 1.78547 | 0.77956 | 0.4464 | 0.4464 | 1.69772 | 0.72803 | 0.4244 | 0.4244 |
| 2 | 1.00591 | 0.32180 | 0.2515 | 0.6978 | 0.96969 | 0.23032 | 0.2424 | 0.6669 |
| 3 | 0.68411 | 0.15961 | 0.1710 | 0.8689 | 0.73938 | 0.14617 | 0.1848 | 0.8517 |
| 4 | 0.52450 | . | 0.1311 | 1.0000 | 0.59321 | . | 0.1483 | 1.0000 |

Appendix Table 2: Percentage of people suffering CHE at various thresholds in 2018

| Percentage share spent on health | Weighted percentage that suffered CHE |
| --- | --- |
| 10 | 43.56 |
| 15 | 31.5 |
| 20 | 23.28 |
| 25 | 18.04 |
| 30 | 13.86 |
| 35 | 10.6 |
| 40 | 7.88 |
